# Renewable Energy and Alternative Fuel Technologies

**DOI:** 10.1155/2015/245935

**Published:** 2015-03-25

**Authors:** Meisam Tabatabaei, Keikhosro Karimi, Rajeev Kumar, Ilona Sárvári Horváth

**Affiliations:** ^1^Biofuel Research Team (BRTeam), Karaj 31438-44693, Iran; ^2^Microbial Biotechnology and Biosafety Department, Agricultural Biotechnology Research Institute of Iran, Karaj 31359-33151, Iran; ^3^Department of Chemical Engineering, Isfahan University of Technology, Isfahan 84156-83111, Iran; ^4^Center for Environmental Research and Technology (CE-CERT), Bourns College of Engineering, University of California, Riverside (UCR), Riverside, CA 92507, USA; ^5^Swedish Centre for Resource Recovery, University of Borås, 501 90 Borås, Sweden

In recent years, biofuels have drawn considerable attention as clean and renewable source of energy. The most attractive types of biofuels are biogas, bioethanol, biodiesel, and biobutanol. Some of these biofuels are likely to play roles in the production of clean energy carriers as promising alternatives to fossil fuels and bring about environmental benefits globally.* Biogas* that primarily contains biomethane is produced through anaerobic digestion of organic wastes. Among the biofuels production processes, biogas process seems to be the easiest to conduct as it does not need sterilization, can be produced in simple reactors at moderate conditions using a natural consortium of microorganisms available in nature such as manure, and does not need a complicated separation and purification process. However, it is more complicated than it appears at first glance specially when high biogas yield is targeted. In fact, the microbiology and biochemistry of biogas production are the most complicated systems compared to those of the other biofuels, as four different processes, that is, hydrolysis, acidogenesis, acetogenesis, and methanogenesis are performed parallel where different types of microbes as a consortium work together. Furthermore, the substrates used for biogas production are a mixture of different components with different degradation properties. Main feedstocks are solid wastes, for example, agricultural, municipal, and food industrial wastes, and wastewater, for example, industrial and municipal wastewater. Technologies for biogas from municipal wastewater sludge are well developed; however, the recently increasing oil prices, unclear future of fossil fuels availability, and environmental impacts have led to significant interest in biogas from other resources especially from industrial and solid wastes. A number of potential substrates, for example, municipal solid waste and manure mixed with bedding materials, have a high potential; however, they contain lignocelluloses that are not easily bioconvertible. Therefore, a number of recent research activities are focused on the improvement of biogas from recalcitrant substrates, for example, lignocelluloses, and high-rate systems for biogas production.

*Ethanol*, the leading liquid biofuel, is widely used for transportation. Currently, sugarcane in Brazil and starchy materials, for example, corn in USA and wheat in Europe, are the main feedstocks (referred to as 1st generation), while lignocellulosic materials in the last decade (2nd generation or lignoethanol) and more recently algal biomass (3rd generation) are suggested as the raw materials. Lignoethanol seems to be the most promising type for the near future as these feedstocks are abundant and available at low prices. However, lignocelluloses are recalcitrant in nature and their processing is more complicated; the process needs a pretreatment step which is still challenging and consumption of hydrolytic enzymes should be minimized. Both of these problems are the subject of a high number of recent investigations. All in all, lignoethanol is more expensive than the 1st generation ethanol and process integration and biorefinery concepts are proposed to make the 2nd generation ethanol competitive.

*Biodiesel* is a promising alternative to diesel fuel, as it has a number of advantages including high cetane number, flash point, and inherent lubricity, creates less exhaust emissions, and contains no polluting chemicals like sulfur, as well as being renewable, biodegradable, and compatible to the existing fuel distribution infrastructure. The process of biodiesel production at industrial scale is developed rather well and the most important challenging and limiting issue in biodiesel production is feedstock supply. In fact, the available feedstock is limited and accounts for over 70% of the total biodiesel production cost. “Crime against humanity” term is raised when edible sources started to be used. Therefore, the future sustainability of this industry is heavily dependent on nonedible feedstock supply and achieving more innovative, integrated, and efficient processes.

*Biobutanol* is considered more advanced compared to the other existing biofuels. Acetone production via acetone-butanol-ethanol (ABE) fermentation is an old process widely established during the First World War for military purposes in the Union of Soviet Socialist Republics, England, Canada, USA, Japan, China, and South Africa. Recently, ABE process attracted a high interest for the production of butanol as a renewable fuel. Main feedstocks used in the old processes were sugar-based substrates such as molasses and starchy materials such as wheat. Recently, low cost lignocellulosic wastes are suggested for ABE fermentation (lignobutanol); however, it suffers from the same problems as in lignoethanol. Furthermore, microorganisms used for biobutanol, for example,* Clostridium acetobutylicum* and* C. beijerinckii,* are more sensitive to inhibitors than the microorganisms used for biogas and ethanol production. Generally, the process of biobutanol production is more complicated than those of ethanol and biogas, as the microorganisms are strictly anaerobic; butanol-producing bacteria are severely inhibited by the process products especially butanol, and separation of products is more energy demanding and complicated. Compared to lignoethanol, lignobutanol is in its preliminary stage of research and a number of problems should be addressed in laboratory and pilot scales before the process becomes competitive to other biofuels at commercial scale.

*Meisam Tabatabaei* *Keikhosro Karimi* *Rajeev Kumar* *Ilona Sárvári Horváth*

